# A novel approach for the generation of genetically modified mammary epithelial cell cultures yields new insights into TGFβ signaling in the mammary gland

**DOI:** 10.1186/bcr2728

**Published:** 2010-10-13

**Authors:** Ethan A Kohn, Zhijun Du, Misako Sato, Catherine MH Van Schyndle, Michael A Welsh, Yu-an Yang, Christina H Stuelten, Binwu Tang, Wenjun Ju, Erwin P Bottinger, Lalage M Wakefield

**Affiliations:** 1 Laboratory of Cancer Biology and Genetics, Center for Cancer Research, National Cancer Institute, 37 Convent Drive MSC 4255, Bethesda MD 20892, USA; 2 Department of Internal Medicine, University of Michigan, 1150 West Medical Center Drive, Ann Arbor, MI 48109, USA; 3 Division of Nephrology, Department of Medicine, Charles R Bronfman Institute for Personalized Medicine, Mount Sinai School of Medicine, 1468 Madison Avenue, New York, NY 10029, USA

## Abstract

**Introduction:**

Molecular dissection of the signaling pathways that underlie complex biological responses in the mammary epithelium is limited by the difficulty of propagating large numbers of mouse mammary epithelial cells, and by the inability of ribonucleic acid interference-based knockdown approaches to fully ablate gene function. Here we describe a method for the generation of conditionally immortalized mammary epithelial cells with defined genetic defects, and we show how such cells can be used to investigate complex signal transduction processes using the transforming growth factor beta (TGFβ)/Smad pathway as an example.

**Methods:**

We intercrossed the previously described *H-2Kb*-tsA58 transgenic mouse (Immortomouse), which expresses a temperature-sensitive mutant of the simian virus-40 large T-antigen (tsTAg), with mice of differing Smad genotypes. Conditionally immortalized mammary epithelial cell cultures were derived from the virgin mammary glands of offspring of these crosses and were used to assess the Smad dependency of different biological responses to TGFβ.

**Results:**

IMECs could be propagated indefinitely at permissive temperatures and had a stable epithelial phenotype, resembling primary mammary epithelial cells with respect to several criteria, including responsiveness to TGFβ. Using this panel of cells, we demonstrated that Smad3, but not Smad2, is necessary for TGFβ-induced apoptotic, growth inhibitory and epithelial-to-mesenchymal transition responses, whereas either Smad2 or Smad3 can support TGFβ-induced invasion as long as a threshold level of total Smad is exceeded.

**Conclusions:**

The present work demonstrates the practicality and utility of generating conditionally immortalized mammary epithelial cell lines from genetically modified Immortomice for detailed investigation of complex signaling pathways in the mammary epithelium.

## Introduction

Transforming growth factors beta (TGFβs) are widely expressed cytokines that play complex roles in both normal physiology as well as pathological states [1,2]. In the mammary gland, TGFβs and their cognate receptors are expressed throughout the development of the gland, where they maintain ductal morphogenesis and architecture, regulate stem cell populations, influence epithelial proliferation and differentiation in response to hormonal cues, and induce apoptosis in the involuting gland (reviewed in [3-5]). These activities are important for maintenance of homeostasis in the normal mammary gland. Indeed, reduction of TGFβ signaling in the mammary gland has been associated with inappropriate differentiation and accelerated tumorigenesis in numerous models, and reduced expression of TGFβ receptors in breast cancer patients correlates with disease progression (reviewed in [5-7]). Paradoxically however, high levels of TGFβ are often detected in advanced human breast cancer, and many preclinical studies have demonstrated that TGFβ can promote metastasis in late-stage disease, through direct effects on the tumor cell such as enhanced motility, invasion, and survival, as well as through effects on the tumor stroma, such as regulation of extracellular matrix composition, stimulation of angiogenesis and suppression of immunosurveillance (reviewed in [5-7]). These findings demonstrate important and complex roles for TGFβ in the normal and diseased mammary gland, and reveal a strong need to better understand the mechanisms by which TGFβ regulates these varied responses.

Canonical signaling by TGFβs is activated by binding of the ligands to cell surface receptors, which then phosphorylate the receptor-activated Smad (R-Smad) proteins, Smad2 and Smad3 [8]. The R-Smads generally partner with a common mediator Smad (Smad4) and translocate to the nucleus, where they regulate gene transcription. Smad2 and Smad3 share a very high degree of homology, with 92% identity at the amino acid level. Genetic knockout studies have revealed a critical role for Smad2 in embryogenesis, whereas Smad3 null mice are viable and survive until adulthood [9]. These distinct phenotypes could represent differing expression patterns rather than intrinsically different activities, however, as insertion of Smad3 into the Smad2 locus is sufficient to rescue lethality in Smad2 null mice [10].

More definitive evidence for distinct biological activities of the two Smads comes from a number of studies. Transcriptome analysis in mouse embryo fibroblasts treated with TGFβ revealed that Smad3 appeared to be the dominant transcriptional regulator downstream of the TGFβ receptor, and that Smad2 functioned primarily in a transmodulatory fashion [11]. Targeted genetic knockout studies have also indicated distinct roles for the two Smads in epithelial homeostasis and response to injury in the liver [12] and the skin [13]. Importantly, it is apparent that different cell types can show different Smad requirements for a given biological response. For example, the cytostatic response to TGFβ is lost in Smad2 null mouse embryo fibroblasts [14], but is enhanced in the HaCAT human keratinocyte cell line following siRNA-mediated knockdown of Smad2 [15]; and the inhibitory effect of TGFβ on T-cell proliferation requires Smad3, while the inhibitory effect on B-cell proliferation does not [16]. Smad utilization thus appears to be contextual and must be studied specifically in the cell type of interest.

Given the complex dual role of TGFβ in breast cancer tumorigenesis, and the desire to generate TGFβ pathway antagonists that might selectively block pro-progression and not tumor suppressor activities of TGFβ, we wished to determine whether the two TGFβ R-Smads contribute differentially to these two classes of activity in the normal and transformed mammary epithelium. Since gene knockdown by RNA interference approaches can never fully ablate the target protein, complete genetic inactivation is necessary to definitively show a requirement for the protein of interest in a given biological response, and this can only be achieved in the mouse. Unlike the situation with human breast epithelium, however, only a small number of mammary cells can be obtained from one mouse, and typically these cells can only be propagated for a few days *in vitro *before undergoing apoptosis [17], posing a challenge for detailed molecular and biochemical analyses.

In 1991 Jat and colleagues generated a mouse (the Immortomouse) that transgenically expressed a temperature-sensitive form of the SV40 Large T antigen (tsTAg) from a broadly-expressing MHC antigen promoter [18]. Conditionally immortalized cells from this model can be expanded and propagated under permissive conditions (33°C with IFNγ) and reacquire many properties of primary cultures under semipermissive or nonpermissive conditions (37°C or 39°C without IFNγ). This approach has been exploited successfully in the study of many rare cell types, including epithelial cells from the cochlea [19] and proximal convoluted tubule of the kidney [20]. In the present study, we have crossed the Immortomouse with mice of differing Smad genotypes to generate a panel of conditionally immortalized mammary epithelial cells (IMECs) that allow us to cleanly dissect the role of the two Smads in different biological responses to TGFβ. This approach has generated interesting biological insights, and should also be broadly applicable to the study of other signal transduction pathways in the mammary epithelium.

## Materials and methods

### Generation of IMEC cultures of different Smad genotypes

**Figure 1:**
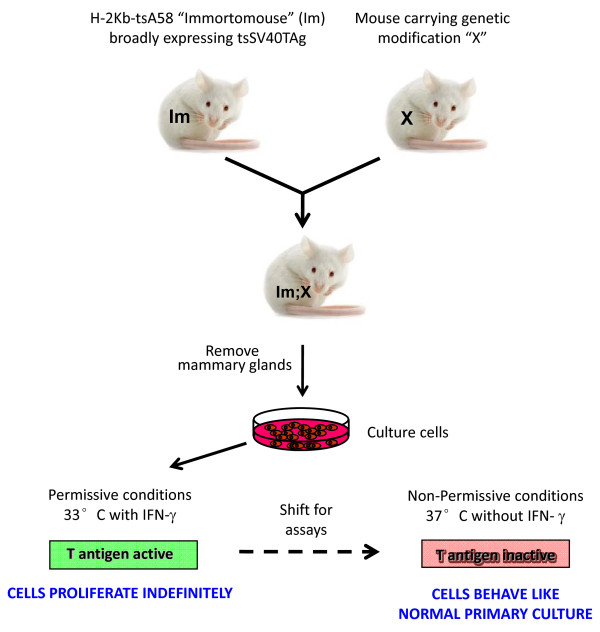
**Using the Immortomouse in combination with genetically modified mice to generate conditionally immortalized cell cultures**. General scheme for breeding strategy, generation and propagation of conditionally immortalized cells.

A schematic for the overall strategy for IMEC generation is shown in Figure 1. The *H-2Kb*-tsA58 transgenic Immortomouse was obtained from Charles River Laboratories (Wilmington, MA, USA). Smad3DelEx8 mice in which the Smad3 gene is constitutively inactivated by insertion of a neomycin cDNA in exon 8 were obtained from Chuxia Deng (National Institute of Diabetes, Digestive and Kidney Diseases, Bethesda, MD, USA) [16], and Smad2LoxPEx2 mice in which exon2 is floxed for conditional deletion were generated as described previously [12]. All animal studies were performed under approved protocols in compliance with the National Cancer Institute's Animal Care and Use Committee guidelines for the ethical treatment of animals.

Mice were intercrossed to generate offspring that had the Immortomouse transgene together with various combinations of Smad mutant alleles. Conditionally IMECs were prepared from 12-week-old virgin mice. Briefly, the number four and number nine (inguinal) mammary glands were aseptically removed and minced with scalpel blades in Ham's F-12 medium supplemented with 10% fetal bovine serum (FBS), 5 μg/ml insulin, 10 ng/ml epidermal growth factor, 100 units/ml penicillin, 100 μg/ml streptomycin, and 1 mg/ml collagenase (blend L; Sigma-Aldrich, St. Louis, MO, USA). Following overnight incubation in this medium at 37°C, the mixture was centrifuged and the fat was removed. The remaining pellet was washed four times in collagenase-free medium and then resuspended in fresh medium and incubated at 37°C. After 4 hours, the medium containing the epithelial cells was transferred to a new dish; the fibroblasts, which attach to the plate much more quickly, were left behind. The following day, the medium containing dead cells and other nonattached debris was removed and fresh medium was added. After 3 days, numerous epithelial colonies could be observed. Some fibroblast contamination was also present; these cells were selectively removed by light trypsinization (0.25% trypsin in 1 mM ethylenediamine tetraacetic acid; Invitrogen, Carlsbad, CA, USA) for 5 minutes at room temperature. The epithelial culture was trypsinized, pipetted up and down to generate a single cell suspension, centrifuged and resuspended in fresh media containing 30 units/ml IFNγ to stimulate the MHC promoter driving tsTAg expression, and incubated at 33°C, the permissive temperature for the tsTAg. After passage through a partial crisis at 7 days, during which ~50% of the cells die, the culture could be stably maintained with minimal further cell death.

For expansion and continuous propagation, cells were grown at 33°C (5% carbon dioxide) in Ham's F-12 media supplemented with 10% FBS, 10 ng/ml epidermal growth factor, 5 μg/ml insulin, 1 μg/ml hydrocortisone, 5 ng/ml cholera toxin, 50 μg/ml gentamycin (complete media) and 30 units/ml IFNγ. For experiments, cells were plated and incubated at 37°C (5% carbon dioxide) in the absence of IFNγ for 2 to 4 days prior to the assay to allow for decay of the tsTAg protein.

For *ex vivo *excision of floxed Smad2, cells were transduced with Adeno-Cre virus (Ad5-CMV-Cre), which was purchased from the University of Iowa Gene Transfer Vector Core (Iowa City, IA, USA). For Smad add-back experiments, adeno-LacZ, Adeno-Smad2 and Adeno-Smad3 were obtained from the late Dr Anita Roberts (National Cancer Institute, Bethesda, MD, USA).

### Immunofluorescence

To immunostain for vimentin and cytokeratins, IMECs were plated in complete media at a density of 5,000 cells/well in 24-well plates. After the cell culture reached 70 to 80% confluence, the medium was removed and cells were washed three times with PBS. Cells were then fixed with methanol for 10 minutes at -20°C and blocked with 0.5% casein for 1 hour with agitation at room temperature. Then cells were dual stained with mouse anti-vimentin (1:200; Sigma) and guinea pig anti-keratin (1:100; Sigma) at 4°C overnight. The following day, cells were washed with PBS and incubated with rabbit anti-guinea pig tetramethyl rhodamine isothiocyanate (Sigma) and horse anti-mouse fluorescein isothiocyanate (Vector Laboratories, Burlingame, CA, USA) secondary antibodies for 1 hour at room temperature.

Cell nuclei were stained with 4',6-diamidino-2-phenylindole. Swiss 3T3 cells were used as a positive control for fibroblasts. To immunostain for E-cadherin and F-actin, cells were plated into an eight-well chamber slide (Nunc Labtek, Thermo Fisher Scientific, Waltham, MA, USA). Cells were fixed with 4% paraformaldehyde for 15 minutes and then permeabilized by 0.1% TritonX-100/PBS (Sigma) for 10 minutes at room temperature. Cells were stained with rat anti-uvomorulin/E-cadherin antibody (clone DECMA-1, 1:100; Sigma) or phalloidin (Alexa Fluor 594, 1:100; Invitrogen) at 4°C overnight. The next day, cells were washed with PBS and cells stained for E-cadherin were incubated with goat anti-rat IgG (Alexa fluor 555, 1:500; Invitrogen) secondary antibody for 1 hour at room temperature. Cell nuclei were stained with 4',6-diamidino-2-phenylindole. Images were acquired a Nikon Eclipse E800 fluorescent microscope (Nikon Instruments Inc., Linthicum Heights, MD, USA) with a Roper Photometrics Cool SNAP fx camera (Roper Scientific GmbH, Ottobrun, Germany) and IPLab 4.0.8 software (BD Biosciences, Franklin Lakes, NJ, USA).

### Quantitative RT-PCR and western blots

For analysis of SV40 T-antigen expression, IMECs were seeded in 100 mm^2 ^culture plates in either permissive conditions (33°C with IFNγ) or nonpermissive conditions (37°C without IFNγ). After 4 days in culture, RNA or protein was isolated and tsTAg expression was analyzed by RT-PCR or western blotting, respectively. For analysis of epithelial-to-mesenchymal transition (EMT) markers, cells were grown under the nonpermissive conditions in complete medium for 24 hours, then switched to medium with reduced serum (0.5% FBS) for a further 18 to 24 hours and, finally, were treated with 2 ng/ml TGFβ for 48 hours prior to isolation of RNA and protein. For all gene targets, total RNA was isolated using the RNeasy kit (Qiagen Inc, Valencia CA, USA) according to the manufacturer's instructions. cDNA synthesis was carried out using M-MLV reverse transcriptase (Invitrogen). The primer sequences for tsTAg are as follows: forward, 5'-GGTGTAAATAGCAAACAAGCAAG-3'; and reverse, 5'-GAATGGGAGCAGTGGTGGAATG-3'. All quantitative RT-PCR data were normalized to cyclophilin A (PP1A) as an internal control for each sample.

For western blot analysis of phospho-Smads, cells were treated as specified for analysis of EMT markers above, except that protein lysates were harvested after 30 minutes of TGFβ treatment. For western blots of tsTAg, lysates were harvested at various timepoints. Total protein (40 μg) was electrophoresed on 4 to 20% Tris-glycine gels and transferred to polyvinylidene fluoride membranes. Membranes were blocked with 5% bovine serum albumin (for anti-Smad2) or 5% nonfat dry milk (for all other antibodies) in Tris-buffered saline with Tween for 45 minutes at room temperature, and were then incubated with primary antibody solutions overnight at 4°C. The primary antibodies used were as follows: anti-Smad2 (Zymed Laboratories Inc., San Franscisco, CA, USA), anti-phospho-Smad2 (Cell Signaling Technology Inc., Danvers, MA, USA), anti-Smad3 (Abcam, Cambridge, MA, USA), anti-phospho-Smad3 (Epitomics Inc., Burlingame, CA, USA), anti-β-actin (Sigma) and anti-tsTAg (Oncogene Research Products, La Jolla, CA, USA). All were used at a 1:1,000 dilution. Secondary antibodies (anti-rabbit, 1:2,000; anti-mouse, 1:8,000) conjugated to horseradish peroxidase were applied for 45 minutes at room temperature, followed by incubation with chemiluminescent reagent (Super Signal; Thermo Scientific Pierce Protein Research Products, Rockford, IL, USA) and exposure to autoradiography film (Eastman Kodak Co., Rochester, NY, USA).

### Gelatin zymography

Following 48 hours of treatment with 2 ng/ml TGFβ in medium containing 0.5% FBS, cell culture supernatants were collected and centrifuged at 400 × *g *for 5 minutes. Cell-free culture supernatants were collected, mixed with Brij-35 (final concentration 0.02%), and stored at -20°C until further use. For zymography, samples were mixed with 6× sample buffer and electrophoresis was performed using precast zymography gels (10% polyacrylamide, 0.1% gelatin; Invitrogen). Proteins were renatured with Renaturing Buffer (Invitrogen) twice for 15 minutes, and zymograms were developed in Developing Buffer (Invitrogen) for 72 hours at room temperature. Gelatinase activity was visualized by staining gels with Coomassie Brilliant Blue G250 (0.25% Coomassie Brilliant Blue G250, methanol 30%, acetic acid 10%) and destained with acetic acid/methanol/dH_2_O (1:3:6). Gels were imaged using a flatbed scanner.

### Flow cytometry

IMECs were grown to ~80% confluence in complete media at 37°C. After harvesting by trypsinization, cells were fixed and permeabilized in a Cytofix/Cytoperm solution (Becton Dickinson, Franklin Lakes, NJ, USA), washed with permeabilization/wash solution (Becton Dickinson), and incubated with primary antibody (anti-cytokeratin 8; Developmental Studies Hybridoma Bank, Iowa City, IA, USA; and anti-cytokeratin 14; Covance, Princeton, NJ, USA) for 16 hours at 4°C. Following washes and incubation with secondary antibodies for 45 minutes at room temperature, cells were re-suspended in staining buffer and analyzed by flow cytometry using a FACSCaliber(tm) instrument (BD Biosciences, San Jose, CA, USA) with FlowJo software (Treestar, Ashland, OR, USA).

### Growth inhibition and apoptosis assays

To determine effects of TGFβ on growth inhibition, IMECs were plated in 24-well tissue culture plates at a density of 15,000 cells/0.5 ml/well in complete media and were shifted from the permissive temperature to 37°C. On day 3, culture medium was changed to Ham's F-12 medium containing 1% FBS and insulin (5 μg/ml) to suppress apoptosis. Recombinant human TGFβ1 (R&D Systems, Minneapolis, MN, USA) was added to cells at various concentrations and incubated for 24 hours. Tritiated thymidine (0.5 μCi) was then added to wells for an additional 4 hours. Cells were then washed, trypsinized and transferred to a filter mat using a Cell Harvester. [^3^H]Thymidine incorporation was assessed using a 1450 Micro-B scintillation counter (Perkin Elmer, Waltham, MA, USA). To assess apoptosis, IMECS were plated in a 96-well plate at a density of 4,000 cells/0.2 ml/well in complete medium. Two days later, culture medium was changed to Ham's F-12 containing 0.2% FBS, and cells were treated with different concentrations of TGFβ for 24 hours. Apoptosis was then measured using the Cell Death Detection ELISA kit (Roche Applied Science, Indianapolis, IN, USA), which measures DNA-histone complexes that are generated during apoptotic cell death.

### Cell migration and invasion

Cell migration and invasion assays were carried out using the Transwell^® ^System (8 μm; BD Biosciences, San Jose, CA, USA). Transwell^® ^inserts are uncoated for migration assays, and are coated with Matrigel(tm) for invasion assays. Briefly, 25,000 IMECs (migration) or 50,000 IMECs (invasion) were plated in complete medium in the top chamber; the bottom chamber was also filled with complete medium. After allowing the cells to attach for 3 to 4 hours, TGFβ was added to both chambers of each well. Plates were incubated for 2 days, and cells that remained in the top chamber were removed. The membranes were then fixed, stained with hematoxylin and mounted on microscope slides, and the number of cells in 10 high-powered fields (40× objective) per membrane was visually quantified.

### Lactogenic differentiation assay

Cells were grown to near-confluence at the nonpermissive temperature and then switched to fresh growth medium containing charcoal-stripped serum and a lactogenic hormone cocktail (5 μg/ml insulin, 1 μM dexamethasone and 1 μg/ml ovine prolactin). The medium was changed daily. Cells were harvested after 72 hours of hormone exposure and were assessed for expression of the milk protein casein by western blot analysis of cell lysates. In some experiments, cells were grown in collagen-coated dishes and exposed to lactogenic hormones for up to 12 days. Similar results were obtained, however, under both sets of conditions.

### Transplantation into the mammary fat pad

All animals were maintained according to the National Cancer Institute's Animal Care and Use Committee guidelines, under approved animal study protocols. The inguinal fat pads of 3-week-old mice were cleared of endogenous epithelium as described previously [21], and several different inocula of IMECs, ranging from 2.5 × 10^4 ^to 2 × 10^6^, were implanted into the cleared fat pads. Each recipient received IMECs in one fat pad and a medium control in the contralateral fat pad. After 10 weeks, the transplanted tissue was harvested for whole-mount analysis. The transplanted glands were removed and spread on a glass slide. After fixation for 2 to 4 hours in Carnoy's solution, glands were hydrated and stained with Carmine alum, dehydrated and mounted as described previously [22]. Whole mounts were directly imaged with a CCD camera mounted on a Zeiss ICM405 microscope (Carl Zeiss Inc, Thornwood, NY, USA).

## Results

### Generation and characterization of conditionally IMECs derived from the Immortomouse

**Figure 2:**
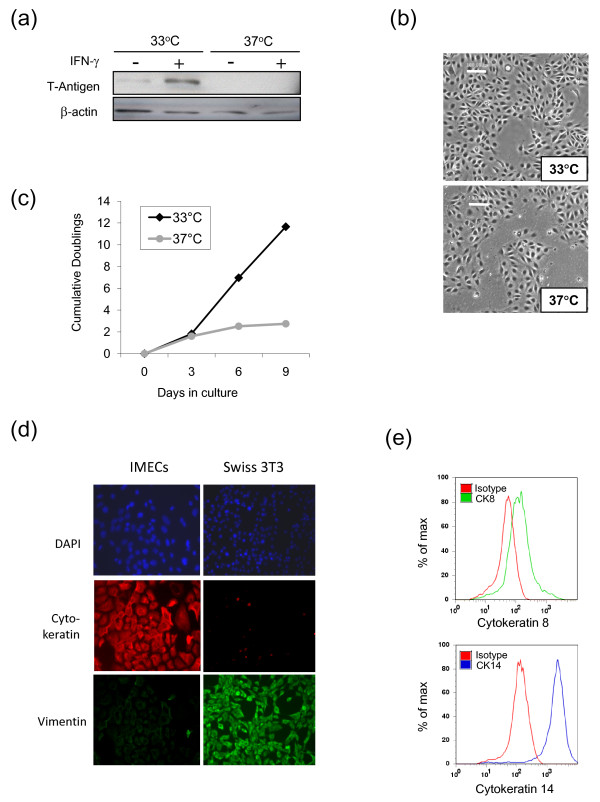
**Morphology, proliferative capacity and intermediate filament expression of immortalized mammary epithelial cell cultures**. **(a) **Western blot of tsTAg expression in immortalized mammary epithelial cells (IMECs) grown in the presence or absence of added IFNγ at permissive (33°C) or nonpermissive (37°C) temperatures as indicated. β-actin used as a loading control. **(b) **Phase contrast images of wildtype IMECs showing a cobblestone epithelial morphology under both permissive and nonpermissive conditions. White scale bar = 100 μm. **(c) **Proliferation of IMECs under the permissive and nonpermissive growth conditions, showing rapid loss of proliferative capacity under nonpermissive conditions. Results representative of two independent experiments. **(d) **Wildtype IMEC cultures were grown under nonpermissive conditions and the expression of intermediate filament proteins was assessed by immunofluorescence using pancytokeratin or vimentin antibodies. Swiss 3T3 fibroblasts were used as a positive control for vimentin expression. DAPI, 4',6-diamidino-2-phenylindole. **(e) **Flow cytometric analysis for expression of cytokeratins CK8 and CK14 in wildtype IMECs grown under nonpermissive conditions.

In contrast with previous published experience using this approach [23], we were able to readily generate propagatable mammary epithelial cell lines from Immortomice for all our genotypes of interest. When initially put into culture, the IMECs grew in epithelioid colonies, and a significant fraction (~50%) of the cells underwent cell death after 6 to 9 days in culture under permissive growth conditions (33°C with IFNγ), as evidenced by slow culture expansion and floating debris. Thereafter, the cultures stabilized and could be propagated with little additional cell death. As seen in Figure 2a, tsTAg protein was strongly induced by IFNγ at 33°C, but not at 37°C. Upon shifting of cells to 37°C, the tsTAg protein decayed to almost undetectable levels within 48 hours (data not shown). Unlike the conditionally IMEC line that was established from a transgenic mouse expressing the tsTAg from a β-lactoglobulin (BLG) promoter (BLG-tsTAg) [23], our IMECs showed a cobblestone epithelial morphology at both permissive and nonpermissive temperatures (Figure 2b), which was maintained over all passages examined (up to passage 60). While cells proliferated continuously at 33°C, on shifting to 37°C the growth rate of the culture slowed significantly after day 3, with no further expansion of the culture after day 6 (Figure 2c). This rapid loss of proliferative capacity is also a feature of primary mammary epithelial cultures. The following experiments were therefore all performed within 5 days of shifting to the nonpermissive conditions.

Consistent with their epithelial origin, the IMEC cultures exhibited strong, culture-wide staining with a pancytokeratin antibody, and were negative for the mesenchymal marker vimentin (Figure 2d). The established cultures are thus indeed epithelial, without fibroblast contamination. Using fluorescence-activated cell sorting analysis, we found that almost all cells were strongly positive for cytokeratin 14 - a basal epithelial marker that is somewhat promiscuously expressed in culture. The luminal cytokeratin 8 was weakly expressed on ~50% of the culture (Figure 2e). It should be noted, however, that mouse mammary epithelial cells have been shown to be much less stable than their human or rat counterparts in their expression of differentiation-specific cytoskeletal markers *in vitro *[24].

**Figure 3:**
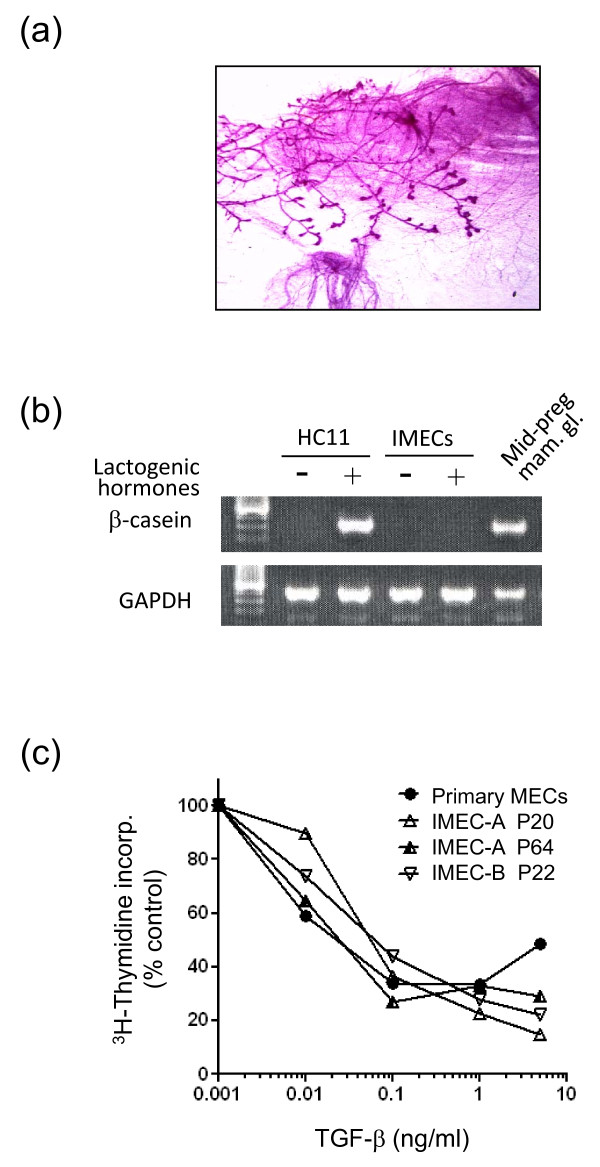
**Functional characterization of immortalized mammary epithelial cell cultures**. **(a) **Whole mount of a reconstituted mammary gland generated by transplantation of 2.5 × 10^4 ^immortalized mammary epithelial cells (IMECs) into a cleared mammary fat pad. **(b) **PCR analysis of β-casein mRNA expression as an indicator of lactogenic differentiation. IMECs growing in two-dimensional culture under nonpermissive conditions were exposed to lactogenic hormones for 3 days prior to isolation of mRNA for analysis. RNA from HC11 cells treated in the same way and RNA isolated from a mid-pregnant mammary gland were used as positive controls. **(c) **Responsiveness to transforming growth factor beta (TGFβ)-mediated growth inhibition in IMECs and primary mammary epithelial cells (MECs) was determined under nonpermissive conditions by assessing incorporation of [^3^H]thymidine. Results are means of triplicate determinations. IMEC-A and IMEC-B are independently generated IMEC cultures derived from different mice, isolated several months apart. The responses of early (P20) and late (P64) passage cultures of IMEC-A were compared.

Following transplantation into the cleared mammary fat pad of nude mice, we observed formation of a mammary ductal tree in 1/20 implants using between 2.5 × 10^4 ^and 2 × 10^6 ^unsorted IMECs/site (Figure 3a). While the efficiency was low under the conditions used, this result suggests that the IMECs retain some capacity to regenerate a morphologically normal mammary epithelium, presumably due to the presence of a small number of conditionally immortalized stem cells in the culture.

The conditionally IMEC line KIM-2 that was generated from the mid-pregnant gland of the BLG-tsTAg transgenic mouse, and the spontaneously immortalized HC11 line also derived from a mid-pregnant mouse, both have the capacity to functionally differentiate in response to lactogenic hormones *in vitro *[23,25]. Our IMECs were derived from virgin mice and did not undergo lactogenic differentiation (Figure 3b). Derivation of parallel lines from mid-pregnant animals, however, should permit this aspect of mammary biology to be examined *in vitro*.

Primary human mammary epithelial cells are extremely sensitive to growth inhibition by TGFβ [26]. We found that proliferation of primary mouse mammary epithelial cells was also strongly inhibited by TGFβ, and that our IMECs showed an essentially identical growth inhibitory response to TGFβ as the primary mouse mammary epithelial cells (Figure 3c). This was true for two independent IMEC isolates, and the property was stable for over 60 passages in culture. The IMECs therefore represent a viable and stable model in which to examine TGFβ responses.

### Generation of IMECS of different Smad genotypes

To delineate the roles of Smad2 and Smad3 in the mammary epithelium, we intercrossed the Immortomouse with two different genetic models of Smad deletion. To address requirements for Smad3, the germline knockout Smad3DelEx8 mouse was used [16]. For Smad2, we used a Smad2 conditional knockout mouse in which exon2 is flanked with loxP sites [12]. We thus generated IMECS of the following four genotypes: wildtype, Smad3^-/-^, Smad2^fl/fl^, and Smad3^-/-^;Smad2^fl/fl^.

**Figure 4:**
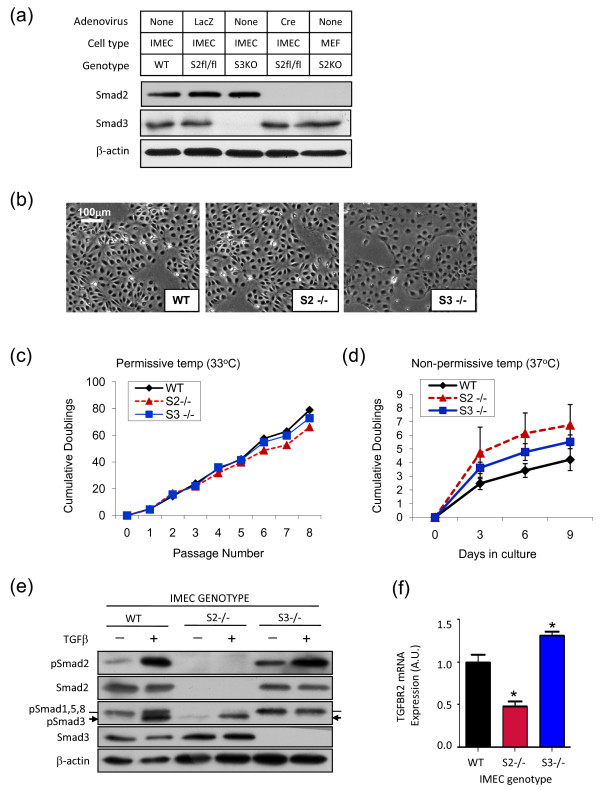
**Characterization of immortalized mammary epithelial cell cultures with different Smad genotypes**. **(a) **Western blot analysis of Smad2 and Smad3 expression in different immortalized mammary epithelial cell (IMEC) cultures. Smad2 was excised from IMECs that were homozygous for the conditional allele of Smad2 (Smad2^fl/fl^) by *ex vivo *exposure to adenovirus expressing Cre recombinase. Adenovirus expressing LacZ was used as a control for nonspecific effects of viral transduction. Mouse embryo fibroblasts (MEFs) derived from germline Smad2 knockout mice (S2KO) were used as a positive control for complete Smad2 deletion. β-actin used as a loading control. WT, wildtype. **(b) **Morphology of Smad null cultures under nonpermissive conditions. White scale bar = 100 μm. **(c) **Long-term growth curves for the various IMEC cultures grown under permissive conditions. Growth plotted as cumulative population doublings. Results are representative of two independent experiments. **(d) **Short-term growth curves for the various IMEC cultures after shifting to nonpermissive conditions. Results are the mean ± standard error of the mean for three independent experiments. **(e) **Western blot of Smad phosphorylation in response to transforming growth factor beta (TGFβ) (2 ng/ml for 30 minutes) in the IMECs of different genotypes. Note that the phospho-Smad3 antibody also recognizes phospho-Smad1, phospho-Smad5 and phospho-Smad8, which comprise the upper of the two bands on the phospho-Smad3 blot. **(f) **Quantitative RT-PCR assessment of TGFβ receptor type II (TGFBR2) mRNA. Data are normalized to the wildtype genotype. *P < 0.05 for expression difference between specified genotype and wildtype control (Student's *t *test). AU, arbitrary units.

Prior to excision of the floxed alleles, Smad2^fl/fl ^IMECs behaved identically to wildtype IMECs and were used interchangeably in some experiments. To generate Smad2 null IMECs, Smad2 was excised from IMEC cultures by *ex vivo *incubation with adenovirus expressing the *Cre *recombinase, and subsequent experiments were carried out either with bulk culture or with clonal isolates; similar results were obtained with both. Western blot analysis confirmed that efficient excision of the floxed Smad2 allele was obtained by this approach (Figure 4a). The wildtype, Smad2 null and Smad3 null cells were morphologically similar in culture, with the exception that colonies formed by Smad3 null cells generally had tighter, more defined borders (Figure 4b). Cultures in which either Smad2 or Smad3 were deleted showed similar growth characteristics to wildtype cultures at the permissive temperature (Figure 4c), while at 37°C Smad2 null cells and Smad3 null cells proliferated slightly faster than wildtype cells, but proliferation rates greatly decreased in all genotypes within a few days (Figure 4d). Interestingly, although double-null IMECs appeared viable following *ex vivo *deletion of Smad2 in Smad3 null cells (data not shown), they could not be further propagated at either permissive or nonpermissive temperatures. This observation suggests that mammary epithelial cells require some level of at least one of the two Smads for continued proliferation. We were therefore unable to perform further studies on the Smad2/3 double-null cells.

We also assessed whether loss of one Smad had any effect on TGFβ signaling through the other Smad. Loss of Smad3 had essentially no effect on TGFβ signaling through Smad2, as assessed by western blot analysis of Smad2 phosphorylation, whereas loss of Smad2 caused a slight reduction in the level of Smad3 phosphorylation by TGFβ (Figure 4e). Consistent with this observation of slightly reduced TGFβ signaling in the Smad2 null cells, we observed a 50% reduction in TGFβ receptor type II mRNA in Smad2 null cells (Figure 4f).

### Smad3, but not Smad2, is required for TGFβ-induced growth inhibition and apoptosis in the mammary epithelium

**Figure 5:**
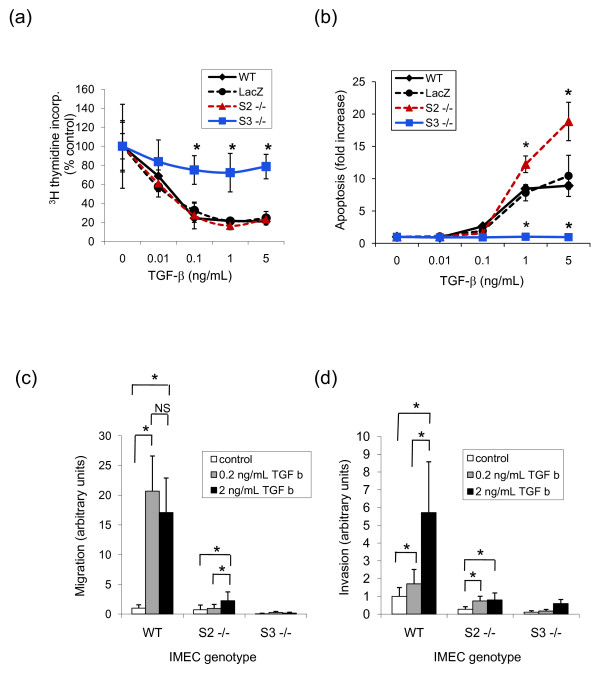
**Smad2 and Smad3 are differentially required for different TGFβ-mediated biological responses**. Immortalized mammary epithelial cells (IMECs) of different Smad genotypes were shifted to nonpermissive temperatures and then assessed for their ability to respond to varying concentrations of transforming growth factor beta (TGFβ) in the following biological assays as described in Materials and methods: **(a) **growth inhibition, **(b) **apoptosis, **(c) **migration, and **(d) **invasion. For the growth inhibition and assays, all data are normalized to the control condition (no added TGFβ) for the respective genotype group. For migration and invasion, data are normalized to the wildtype (WT) IMECs, no added TGFβ control condition. LacZ indicates Smad2^fl/fl ^cells that have been exposed to the LacZ control adenovirus and therefore have a wildtype complement of Smad2 and Smad3. Data are the mean ± standard deviation of three determinations. All experiments were repeated at least twice with essentially the same results. (a, b) *P < 0.05 for the difference between the indicated Smad genotype and wildtype control. (c, d) *P < 0.05 for the difference between specified pairwise comparisons.

TGFβ has tumor-suppressive activity in many normal epithelia, and the inhibition of proliferation and/or the induction of apoptosis are likely to contribute to this activity (reviewed in [27]). We therefore sought to determine whether these activities were dependent upon Smad2, Smad3, or both in the mammary epithelium. Incubation of wildtype IMECs with TGFβ in the presence of insulin, to suppress apoptosis, resulted in potent growth inhibition, with ~80% growth arrest at 1 to 5 ng/ml TGFβ (Figure 5a). IMECs that lacked Smad2 were growth inhibited as efficiently as wildtype IMECs. Loss of Smad3, however, greatly reduced the sensitivity of the IMECS to TGFβ-induced growth arrest. Our previous experiments with primary MECs had suggested that Smad3 was not necessary for growth inhibition by TGFβ [28]. Only a single high concentration of TGFβ was tested in that study, however, and the growth inhibition achieved by the TGFβ was much less extensive, so the dependence on Smad3 was not readily apparent. We have subsequently found the growth inhibitory effect of TGFβ on MECs to be very dependent on culture conditions. In the more physiologically relevant dose range under optimized culture conditions, our current study shows that Smad3 is clearly necessary for a significant growth inhibitory response to TGFβ, while Smad2 is dispensable.

Incubation of wildtype IMECs with TGFβ in the absence of added insulin resulted in a potent induction of apoptosis (Figure 5b), consistent with the known role of TGFβ in driving apoptosis during involution [4]. We have previously shown that Smad3 was required for this response *in vivo*, but we did not address the role of Smad2 [28]. Using the IMECs, we found that deletion of Smad3 completely ablated the ability of TGFβ to induce apoptosis, even at the highest tested concentrations of TGFβ, whereas deletion of Smad2 reproducibly rendered cells nearly twofold more sensitive to induction of apoptosis by TGFβ, when compared with wildtype cells (Figure 5b). Smad2 expression is thus not required for induction of apoptosis in the mammary epithelium, and may actually oppose the apoptosis-inducing effects of TGFβ, whereas Smad3 is absolutely required for this response.

### Knockout of either Smad2 or Smad3 eliminates the induction of cell migration and invasion by TGFβ

As carcinoma cells develop resistance to the tumor suppressive effects of TGFβ in late-stage disease, tumor-promoting biological responses such as the stimulation of cell migration and invasion become more dominant (reviewed in [1]). We found that wildtype IMECs were capable of migrating in Transwell assays in response to TGFβ, exhibiting a 15-fold to 20-fold increase in migration over untreated cells (Figure 5c). The cells also showed a low level of basal invasion through Matrigel that could be induced nearly sixfold by treatment with TGFβ (Figure 5d). Cells that lacked either Smad2 or Smad3, however, lost their ability to migrate or invade, both basally and in response to TGFβ. Therefore, as opposed to the growth inhibition and apoptotic responses for which Smad3 alone was necessary and sufficient, expression of both Smads appears to be required for induction of the migratory and invasive responses by TGFβ.

### Smad2 and Smad3 function interchangeably in the invasion response

**Figure 6:**
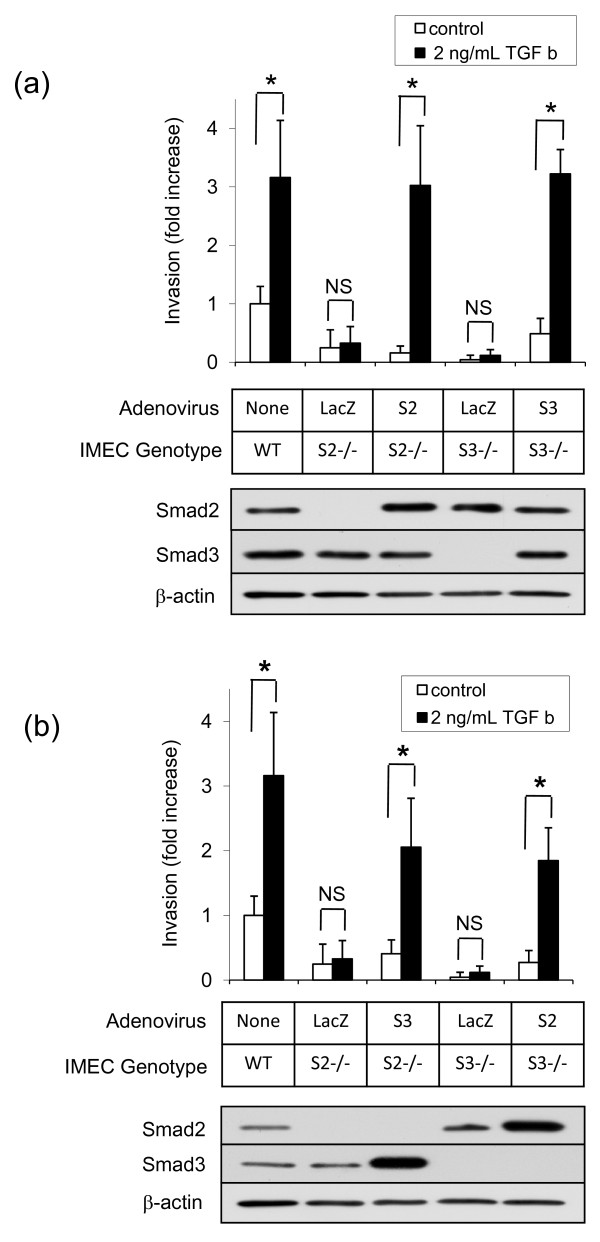
**Exogenous Smad2 or Smad3 expression restores TGFβ-mediated invasion in immortalized mammary epithelial cells lacking Smad**. **(a) **Adenoviral expression of Smad2 in Smad2 null cells, or of Smad3 in Smad3 null cells, restores sensitivity to transforming growth factor beta (TGFβ)-induced invasion. **(b) **Adenoviral expression of Smad3 in Smad2 null cells, or of Smad2 in Smad3 null cells, largely restores sensitivity to TGFβ-induced invasion. All values normalized to wildtype (WT) control values. Results are the mean ± standard deviation of three determinations. *P < 0.05 for the difference between specified pairwise comparisons. Western blot analysis of Smad expression in adenovirally transduced immortalized mammary epithelial cells (IMECs) is shown for each experiment. β-actin used as a loading control. NS, not significant.

A dependence on both Smad2 and Smad3 could either reflect an independent requirement for each Smad to be present, or it might indicate that the two Smads are interchangeable for eliciting the invasion response but that a threshold level of R-Smad must be exceeded for the response to occur. To differentiate these two possibilities, Smad2 or Smad3 were reintroduced into IMECs of the various null genotypes by adenoviral transduction. Ectopic expression of Smad2 in Smad2-deleted cells or Smad3 in Smad3 null cells was sufficient to rescue sensitivity to TGFβ-induced invasion, giving levels of induction that were comparable with those seen in wildtype cells (Figure 6a). This add-back experiment confirms that Smad-deficient cultures were unable to invade because Smad2 and Smad3 are required for this biological response, and not because of some Smad-independent difference in the cell populations due to adaptation to the loss of a Smad. Interestingly, when Smad3 was introduced into Smad2 null IMECs, and Smad2 into Smad3 null cells, the transduced cells regained 60 to 70% of their invasive capacity - suggesting that the two Smads are essentially interchangeable for this biological response, and that a threshold level of expression of either Smad must be exceeded for invasion to occur (Figure 6b).

### Smad dependency of the TGFβ-induced epithelial-to-mesenchymal transition and regulation of metalloproteinases

TGFβ is a strong inducer of EMT in many biological systems, with a variable requirement for the R-Smads depending on the cell type studied [29]. In general, however, TGFβ-induced EMT appears dependent on Smad3, and Smad2 may play an opposing role by maintaining an epithelial morphology under basal conditions [12,30]. When we excised Smad2 *ex vivo *in our IMEC cultures, a very small fraction of the population did spontaneously acquire a spindled appearance (data not shown), and the identity of this susceptible subpopulation will require further study. Unlike the situation with hepatocytes [12], however, the bulk of the Smad2 null IMEC population retained an epithelial morphology in the basal state, although there was some evidence for molecular changes consistent with a partial EMT (see below).

**Figure 7:**
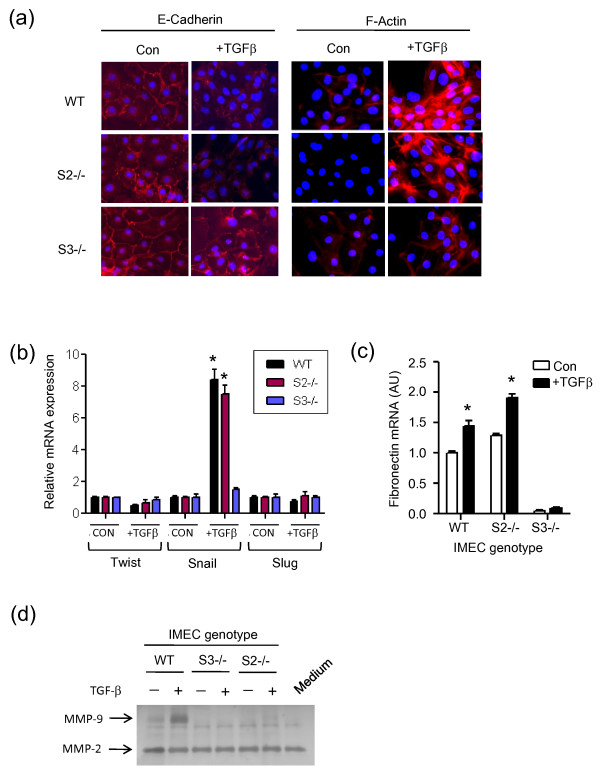
**Smad dependency of induction of molecular markers of epithelial-to-mesenchymal transition and of MMP-9 by TGFβ**. **(a) **Immunofluorescent staining for E-cadherin and F-actin following 48 hours with or without treatment with 2 ng/ml transforming growth factor beta (TGFβ). **(b) **Quantitative RT-PCR determination of mRNA levels for key transcriptional regulators of EMT under the same treatment conditions as (a). All data are normalized to the no TGFβ condition for each genotype. *P < 0.05 for the difference between the TGFβ-treated and control conditions. **(c) **Quantitative RT-PCR for fibronectin mRNA. Data normalized to the wildtype (WT) genotype, no TGFβ treatment. *P < 0.05 for the difference between the TGFβ-treated and control conditions. **(d) **Gelatin zymography of cell-conditioned medium following 48 hours with or without treatment with 2 ng/ml TGFβ. Medium indicates medium alone control; MMP-2 is present in the serum contained in the culture medium.

None of the IMEC lines we developed responded to exogenously added TGFβ with a classic morphological EMT (data not shown). The widely-studied NMuMG mouse mammary epithelial cell line seems to be unusually sensitive to induction of EMT by TGFβ [31], as only a minority of cell lines from a large panel of normal and transformed breast epithelial cell lines showed such a strong response in the 48-hour timeframe [32]. Despite the absence of a morphological EMT, however, we saw molecular evidence suggesting that a partial EMT does occur in response to TGFβ in the wildtype and Smad2 null IMECS, but not in the Smad3 null cells. Furthermore, the Smad2 null IMECs showed some indication of early EMT-related changes in the bulk culture in the untreated state. E-cadherin was localized primarily at cell-cell junctions of untreated cultures of wildtype and Smad3 null cells, although this feature was less clear in the Smad2 null cells (Figure 7a). In response to treatment with TGFβ, E-cadherin was delocalized and partially lost from wildtype and Smad2 null IMECs, but not from Smad3 null IMECs. Similarly, phalloidin staining for F-actin showed a weakly positive cortical distribution in untreated wildtype and Smad3 null cells, which was not readily apparent in Smad2 null cells. Following treatment with TGFβ, both wildtype and Smad2 null cells, but not Smad3 null cells, showed formation of actin stress fibers. This partial induction by TGFβ of features of EMT in the wildtype and Smad2 null cells was supported by the demonstration that TGFβ could induce expression of Snail, a transcriptional regulator of EMT, in wildtype and Smad2 null cells, but not in Smad3 null cells (Figure 7b). In contrast, expression of Twist and Slug was relatively unaffected by TGFβ in all three cell types. Consistent with the induction of some mesenchymal-like properties, TGFβ also induced the upregulation of fibronectin mRNA in wildtype and Smad2 null IMECs, but not in Smad3 null IMECs (Figure 7c).

Therefore, as has been observed in other cell types [12,33,34], the ability of TGFβ to induce features of EMT in IMECs is dependent on the presence of Smad3 but not of Smad2. Since the invasion and migration responses to TGFβ were lost in both Smad2 null and Smad3 null IMECs while the EMT response was only lost in the Smad3 null cells, we searched for a different underlying mechanism to explain the Smad dependency of the invasion response. Invasion is dependent on the induction of metalloproteinases to degrade the extracellular matrix, and TGFβ is known to affect the expression and/or activity of several metalloproteinases [35]. Using gelatin zymography, we showed that the ability of TGFβ to induce MMP-9 is essentially lost in both Smad2 null cells and Smad3 null cells (Figure 7d). The ability of TGFβ to increase MMP-9 activity thus shows the same Smad dependency as the invasion response program, and provides a plausible molecular underpinning for the observed dependency pattern of the invasion response.

## Discussion

Understanding the detailed mechanisms by which various molecular mediators regulate mammary gland biology is of strong interest in the study of both normal physiology as well as disease states such as cancer. In the present article, we have combined the power of mouse genetics to totally ablate a gene of interest, with a conditional immortalization approach that allows us to overcome the challenge of generating sufficiently large numbers of primary mouse mammary epithelial cells of defined genotype for molecular and biochemical analysis. We have applied this approach to address the roles of Smad2 and Smad3 in mediating TGFβ responses in the mammary epithelium. The results have yielded interesting insights into TGFβ signaling and the approach is likely to be broadly applicable to other signaling pathways.

The mammary epithelial cells that we generated by this approach could be propagated essentially indefinitely at the permissive temperature (at least 60 passages), but retained many of the properties of primary cultures when shifted to the nonpermissive temperature, including the epithelial morphology, expression of epithelial cytokeratins, and responsiveness to the growth inhibitory effects of TGFβ. Importantly, only a single female mouse of the desired genotype was required for the isolation and propagation of large numbers of conditionally immortalized cells for downstream molecular and biological analyses. This feature of the approach is particularly useful when multiple genes need to be knocked out simultaneously, as with the Smad3^-/-^;Smad2^fl/fl^;Im mice that had five independently segregating genetically modified alleles.

A related approach to the generation of conditionally IMECs has previously been published, in which the same tsTAg transgene was used, but expression was driven by the BLG milk protein promoter [23]. The MECs generated from the BLG model appeared to show some developmental plasticity and instability, irreversibly developing a spindled non-epithelial morphology when shifted to the permissive temperature, and acquiring the ability to form colonies in soft agar, a characteristic of transformed cells. In contrast, the various IMEC cultures that we have generated from the Immortomouse are phenotypically epithelial under permissive or nonpermissive conditions and have not transformed during the time we have maintained them in culture (up to 60 passages). Furthermore, the BLG promoter is expressed during mid-to-late pregnancy and lactation, restricting generation of conditionally IMECs to those developmental stages. In contrast, the H-2Kb major histocompatibility promoter used in the Immortomouse should be expressed at all developmental stages. In the present study, we derived our mammary epithelial cells from virgin mice, but there is no reason why the same approach could not be used to generate conditionally immortalized epithelial cells from pregnant, lactating, involuting or postinvolution glands to address the molecular context changes and biological features expressed at these different stages. Indeed, since each mouse has multiple mammary glands, it should be possible to derive cells representing different stages of functional differentiation from the same mouse. We therefore feel that use of the Immortomouse complements existing approaches and offers some significant additional advantages.

To illustrate the utility of this approach, we generated a novel panel of conditionally immortalized lines with different Smad genotypes that we then used to assess the relative dependency of different biological responses to TGFβ on the downstream mediators, Smad2 and Smad3. We found distinct patterns of dependency on the two Smads as summarized in Table 1.

**Table 1:** Receptor-activated Smad dependency of different biological responses to TGFβ in mammary epithelial cells

Biological response to TGFβ	Potential role in tumorigenesis	Receptor-activated Smad requirement
Growth inhibition	Tumor suppression	Smad3
Apoptosis	Tumor suppression	Smad3; Smad2 opposes
Migration and invasion	Pro-progression	Smad2 or Smad3; a threshold level of receptor-activated Smad must be exceeded
Epithelial-to-mesenchymal transition	Pro-progression	Smad3; Smad2 opposes basally
Induction of metalloproteinases (MMP-9)	Pro-progression	Smad2 or Smad3

Smad, Sma and MAD (mothers against decapentaplegic)-related protein; TGFβ, transforming growth factor beta.

For TGFβ to induce growth inhibition, apoptosis or a partial EMT in the mammary epithelial cells, we found that Smad3 but not Smad2 was required. Indeed, Smad2 partially opposed the effect of TGFβ in inducing apoptosis, and provided some protection against EMT-like changes in the basal state. This pattern of Smad dependency has also been observed by other researchers in different cell types. Smad3 is the critical mediator of growth inhibitory and proapoptotic responses to TGFβ in primary murine hepatocytes, a mouse mammary epithelial cell line, and a human keratinocyte cell line [12,36,37]. Similarly, Smad3 is essential for TGFβ to induce pathological EMT in the renal tubulointerstitial epithelium and the lens epithelium of the eye [33,34]. The ability of Smad2 to oppose or reduce certain Smad3-dependent responses has also been observed by others, for the growth inhibitory effect of TGFβ [15], for the EMT response [12] and for some transcriptional responses [15,12,38]. Opposing effects of Smad2 and Smad3 on transcriptional regulation of the *goosecoid *gene have been ascribed to competition between Smad3 and Smad4 for binding to the Smad binding element adjacent to the FAST2/Smad2 binding site [38].

A completely different pattern of Smad dependency was observed for TGFβ-induced migration and invasion responses in the mammary epithelial cells. In this present article, we showed that knockout of either Smad2 or Smad3 eliminated these two biological responses, initially suggesting to us that both Smads were required. In add-back experiments, however, we found that overexpression of Smad3 could substitute for loss of Smad2, and *vice versa*, in restoring the invasion response. The invasion response may therefore require a critical threshold level of activated Smad that can be supplied by either Smad2 or Smad3 when present in sufficient quantity. A similar mechanism has also been implicated in early mouse embryo development where one Smad appears to be able to substitute for the other in rescuing some of the developmental phenotypes [10]. We further showed that the ability of TGFβ to induce MMP-9 activity was also lost on ablation of either Smad2 or Smad3, suggesting one possible molecular effector program that might underlie the observed Smad dependency of the invasion response.

The differential Smad requirement for the regulation of different biological responses could be attributable to the differences in the Smad interactomes. Both Smad2 and Smad3 have sizeable interactomes, consisting of many DNA-binding proteins and transcriptional cofactors [39,40]. The majority of these proteins can interact with either Smad2 or Smad3, and the transcriptional programs mediated by these common interactors may underlie those biological responses that are regulated by either/both Smads. There are a number of transcriptional partners that only bind to Smad3, however, such as the C/EBPs, several FoxO family members, ATF3, and certain steroid hormone receptors [39]. This class of interactors may be important for responses that are specifically dependent on Smad3. The requirement of a particular biological response for a given Smad is therefore likely to be highly dependent on the molecular context provided by the spectrum of transcriptional cofactors and modulators that are expressed in a given cell. Genome-wide approaches to identify Smad target genes, such as ChIP-chip and ChIP-seq, will provide important insights into these questions [41,42].

It is tempting to extrapolate from our results on the normal mammary epithelium to suggest that Smad2 might be a better molecular target than TGFβ for breast cancer therapy, since Smad2 appears only to be required for TGFβ responses that might promote tumorigenesis (migration and invasion) and not for potentially tumor suppressive responses (growth inhibition and apoptosis). In the epidermis, however, Smad2 has been shown to have tumor suppressor activity [30]. Furthermore, in rat prostatic epithelial cells, Smad2 was shown to be critical for the proapoptotic effect of TGFβ in a premalignant basal cell line (NRP152), while Smad3 mediated the apoptotic response in a malignant luminal carcinoma cell line (NRP154) derived from the same rat prostate [43]. Together, these data suggest that the requirement for a specific Smad may vary with the nature of the target epithelium, the differentiation state of the cell, and/or the stage of malignant progression. This issue needs to be further addressed in the mammary epithelium. Introducing oncogenic lesions by transgenic breeding strategies or by *ex vivo *transduction of IMECs could provide one approach to this question.

## Conclusions

We have developed an experimental approach that allows the generation and continued propagation of conditionally immortalized mammary epithelial cells from mice with defined genetic lesions. Using the TGFβ pathway as a model, we demonstrated the utility of this strategy by elucidating the Smad dependency of a number of key biological responses to TGFβ. This approach represents a powerful tool for exploring cell biology, and could be readily applied to other signaling pathways and to different stages in mammary gland development and/or malignant progression.

## Abbreviations

BLG: β-lactoglobulin; EMT: epithelial-to-mesenchymal transition; FBS: fetal bovine serum; IFN: interferon; IMEC: immortalized mammary epithelial cell; MMP: matrix metalloproteinase; PBS: phosphate-buffered saline; PCR: polymerase chain reaction; R-Smad: receptor-activated Smad; RT: real-time; siRNA: small interfering RNA; Smad: Sma and MAD (mothers against decapentaplegic)-related protein; SV40: simian virus-40; TGFβ: transforming growth factor beta; tsTAg: temperature-sensitive mutant of the SV40 large T antigen.

## Competing interests

The authors declare that they have no competing interests.

## Authors' contributions

EAK, ZD and LMW developed ideas and designed experiments. EAK and LMW wrote the manuscript. EAK and ZD performed the majority of the experiments, with assistance from CMHVS, MAW and Y-aY. BT, MS, and CHS contributed expertise and helped perform fluorescence-activated cell sorting analyses, immunofluorescence and zymography. WJ and EPB provided intellectual input and expertise, and contributed to breeding of mice with mixed Smad genotypes and manuscript editing.
